# Mitochondrial dynamics involves molecular and mechanical events in motility, fusion and fission

**DOI:** 10.3389/fcell.2022.1010232

**Published:** 2022-10-19

**Authors:** Adam Green, Tanvir Hossain, David M. Eckmann

**Affiliations:** ^1^ Department of Anesthesiology, The Ohio State University, Columbus, OH, United States; ^2^ Center for Medical and Engineering Innovation, The Ohio State University, Columbus, OH, United States

**Keywords:** mitochondria, motility, fusion, fission, mitochondrial DNA, live-cell imaging, disease, therapeutics

## Abstract

Mitochondria are cell organelles that play pivotal roles in maintaining cell survival, cellular metabolic homeostasis, and cell death. Mitochondria are highly dynamic entities which undergo fusion and fission, and have been shown to be very motile *in vivo* in neurons and *in vitro* in multiple cell lines. Fusion and fission are essential for maintaining mitochondrial homeostasis through control of morphology, content exchange, inheritance of mitochondria, maintenance of mitochondrial DNA, and removal of damaged mitochondria by autophagy. Mitochondrial motility occurs through mechanical and molecular mechanisms which translocate mitochondria to sites of high energy demand. Motility also plays an important role in intracellular signaling. Here, we review key features that mediate mitochondrial dynamics and explore methods to advance the study of mitochondrial motility as well as mitochondrial dynamics-related diseases and mitochondrial-targeted therapeutics.

## Introduction

The origins of mitochondria are widely accepted to be traced to an ancestral endosymbiotic bacterium known as alphaproteobacterium (Gray et al., 2001). Phylogenomic analyses have traced the origin of eukaryotes to a group of Archaea, coined Asgards (Spang et al., 2015), and have also confirmed the connection between the mitochondrial endosymbiont and alphaproteobacterial ancestor (Wang and Wu, 2015). The transformation into mitochondria occurred approximately 1.5 billion years ago and altered forms started to appear after the evolutionary paths began to diverge (Mentel et al., 2014). The current human mitochondrial proteome has an estimated 15%–20% relation to the original endosymbiont DNA (Gabaldon and Huynen, 2007). Theories and debates regarding the exact origin are still ongoing.

Mitochondria are commonly known as the “powerhouse” of the cell, but this overly simplistic description ignores many important aspects of these intracellular organelles. Mitochondria are indeed the intracellular site for aerobic respiration and the subsequent synthesis of ATP. Extensive research over the past half a century has revealed numerous biochemical processes involving mitochondria such as protein synthesis, cell metabolism, cell death, calcium signaling, and gene expression in the nucleus that are critically important in the survival of eukaryotes (Mposhi et al., 2017; Vakifahmetoglu-Norberg et al., 2017; Patra et al., 2021).

Mitochondria are not static structures, but in fact they are dynamic. They have the ability to change their morphology through fusion and fission events and they are also motile with the ability to relocate within the cell to meet energy demands. The field of mitochondrial dynamics was founded over a century ago through Lewis and Lewis’s observations with light microscopy showing that “any one type of mitochondria such as a granule, rod or thread may at times change into any other type or may fuse with another mitochondrium [sic], or it may divide into one or several mitochondria.” (Lewis and Lewis, 1914). Technological advancements in microscopy with dyes or targeted fluorescent proteins over the last 30–40 years have made it easier to track mitochondrial movement in live cells (Kandel et al., 2015; Higuchi-Sanabria et al., 2016; Liu et al., 2017). Mitochondrial motility has been most heavily studied in neurons, since neuronal mitochondria travel long distance in axons and dendrites along microtubule tracks *via* motor proteins and adaptors (Ehlers, 2013; Lin and Sheng, 2015). The strategic localization of mitochondria at particular subcellular sites is necessary for energy use and calcium signaling. Thus, neuronal activity regulates the movements of mitochondria across the axonal and dendritic arbors. Mitochondrial transport in neurons is bidirectional with anterograde and retrograde directions (Mandal and Drerup, 2019). Furthermore, movement of neuronal mitochondria is not continuous due to frequent pausing for extended periods of time in sites with high energy demand (Spillane et al., 2013) and with excess of cytosolic calcium, such as synapses (Yi et al., 2004; Zhang et al., 2010). Finally, the moving pattern of mitochondria is wiggly due to mitochondrial dynamics including constant fusion and fission.

Mitochondrial fission is the process through which mitochondria divide into two daughter organelles, whereas mitochondrial fusion is the combining of two separate mitochondria into a larger one. The significance of fission and fusion events was not well characterized or understood until the discovery of mutations in fusion and fission that caused human diseases and correlated strongly with mitophagy and apoptosis (Lin and Beal, 2006; Pieczenik and Neustadt, 2007; Chistiakov et al., 2018). The dynamic behavior of mitochondria requires a balance between fusion and fission in order to maintain equilibrium when responding to constantly changing physiological conditions. In order for this balance to be maintained, mitochondria rely on a number of key fusion and fission proteins for the creation of mitochondrial networks or fragmentation as well as movement within the cell or axon.

In this review we provide an overview of mitochondrial dynamics, including fission, fusion, and motility, while bringing focus to the molecular mechanisms driving these mitochondrial dynamic events as well as the diseases that occur when such mitochondrial dynamics are disrupted. The opportunity for therapeutic interventions based on specific molecular factors is also discussed.

## Proteins involved in fusion and fission

Key proteins involved in mitochondrial fusion and fission are included in Table 1. The machinery that is directly responsible for the important dynamic activity recognized as mitochondrial fission and fusion all belongs to the same family of highly conserved large dynamin-related GTPase proteins (DRPs) that control membrane remodeling events with their ability to hydrolyze GTP and self-assemble (Praefcke and McMahon, 2004). The first-identified member of the DRP family, dynamin, is best studied for its function in clathrin-mediated endocytosis where it mediates scission of the neck of clathrin-coated vesicles from the plasma membrane (Faelber et al., 2013). Mitochondrial fission in mammals is similarly catalyzed by a cytosolic DRP known as dynamin-related protein 1 (*DRP1*) (Smirnova et al., 2001; Praefcke and McMahon, 2004). The yeast ortholog of *DRP1*, Dynamin-1 (*Dnm1*), is also required for mitochondrial fission (Ingerman et al., 2005). The exact mechanism through which *Dnm1* drives membrane constriction and mitochondrial fission events is unclear, but it is postulated that the spiral-like structures that *Dnm1* forms possess dimensions similar to that of mitochondrial constriction sites and drive mitochondrial division (Tieu and Nunnari, 2000; Ingerman et al., 2005).

**TABLE 1:** Mitochondrial fusion and fission regulator proteins.

Mitochondrial fusion and fission proteins	Regulation Type	Regulator protein/mRNA	Fusion/Fission response	References
*MFN2*	Transcription	PGC1a/ERRa; MEF2	Increased Fusion	(Soriano et al., 2006), (Cartoni et al., 2005), Martorell-Riera et al. (2014)
	Translation	miR-761; miR-106b; miR-214	Decreased Fusion	(Zhou et al., 2016), (Wu et al., 2016), Bucha et al. (2015)
	Ubiquitination	PARKIN; MARCH5; HUWE1; MUL1	Decreased Fusion	(Gegg et al., 2010), (Nakamura et al., 2006), (Leboucher et al., 2012), Peng et al. (2016a)
	Phosphorylation	JNK; *PINK1*	Decreased Fusion	(Leboucher et al., 2012), Chen et al. (2013)
*MFN1*	Translation	miR-19b; miR-140	Decreased Fusion	(Li et al., 2014a), Li et al. (2014c)
	Ubiquitination	PARKIN	Decreased Fusion	Gegg et al. (2010)
	Phosphorylation	ERK1/2	Decreased Fusion	Pyakurel et al. (2015)
*OPA1*	Transcription	NF-kB	Increased Fusion	Parra et al. (2014)
	Cleavage	OMA1; YME1L	Decreased Fusion	Anand et al. (2014)
*DRP1*	Translation	miR-30; miR-499	Decreased Fission	(Li et al., 2010), Cereghetti et al. (2008)
	Phosphorylation	PKA; Pim-1; GSK3B	Decreased Fission	(Chang and Blackstone, 2007), (Din et al., 2013), Chou et al. (2012)
		CaMKIa; ROCK1; CDK1; ERK1/2; PKCd	Increased Fission	(Han et al., 2008), (Wang et al., 2012), (Chang and Blackstone, 2007, Santel and Frank, 2008, Kashatus et al., 2015, Serasinghe et al., 2015, Cribbs and Strack, (2007)
	Dephosphorylation	Calcineurin	Increased Fission	(Cribbs and Strack, 2007, Kim et al., 2011, Cereghetti et al. (2008)
	Sumoylation	MAPL	Increased Fission	Zunino et al. (2007)
	Desumoylation	SENP5	Decreased Fission	Zunino et al. (2007)
	Ubiquitination	PARKIN	Decreased Fission	Lutz et al. (2009)
		MARCH5	Increased Fission	Karbowski et al. (2007)
	O-linked N-Acetylglucosamination	N-acetyl-glucosaminidase	Increased Fission	(Gawlowski et al., 2012), Park et al. (2021)
	S-nitrosylation	Nitric oxide	Increased Fission	Cho et al. (2009)

Three proteins have been discovered in yeast to have a direct effect on mitochondrial fusion. Two of these are outer-membrane proteins, fuzzy onions (*Fzo1*) and *Ugo1* and the third is the inner-membrane protein, *Mgm1*. The mammalian orthologs of these proteins are mitofusins 1 (*MFN1*) and 2 (*MFN2*) in relation to *Fzo1* and optic atrophy protein (*OPA1*) in relation to *Mgm1*. The loss of any of the genes for these proteins leads to a disruption in the balance of mitochondrial fission and fusion and results in severe pathophysiological consequences. Mouse models lacking *MFN1*, *MFN2* (Chen H. et al., 2003), *OPA1* (Davies et al., 2007), or *DRP1* (Wakabayashi et al., 2009) are embryonic lethal.

### *Fzo1* and *MFN1* and *MFN2*

A study published in 1997 showcased the *Fzo1* gene, the first known regulator of mitochondrial fusion through the molecular genetic analysis of male *Drosophila melanogaster* (Hales and Fuller, 1997). Mutation in *Fzo1* caused male sterility and defects in mitochondrial fusion leading to the finding that *Fzo1* is required for mitochondrial fusion during spermatogenesis (Hermann et al., 1998; Rapaport et al., 1998). Stemming from this finding was the discovery of the core components of the mitochondrial fusion machinery - *Fzo1* in yeast and its mammalian orthologs *MFN1* and *MFN2*. As DRPs, they were originally characterized as containing a GTPase domain, a middle domain, and a C-terminal assembly or GTPase effector domain (GED) (Koshiba et al., 2004). New insights from structural studies have revised the organization of DRP1 as well as other proteins in the dynamin family (Frohlich et al., 2013). The large N-terminal GTPase domain is followed by the middle domain, a helix bundle region and two transmembrane segments. These are connected by a small and functionally important loop containing amino acids known as the variable domain (VD) in the intermembrane space at the C-terminus. Both the N and C termini of DRPs face the cytosol (Hinshaw, 2000; Chen H. et al., 2003; Eura et al., 2003). Recent information however posits that the C terminus resides in the intermembrane space instead (Mattie et al., 2018). The positions of the termini within the cell have a functional importance as described in the below sections. The positions of the protein termini within the cell have functional importance as will be described in the sections below. There remains need for additional information to be ascertained regarding this discovery in order for there to be full understanding of these structure-function relationships involved in mitochondrial fusion (Giacomello and Scorrano, 2018). *Fzo1* also contains three heptad repeat regions which are likely to be important for inter- and intra-molecular interactions and mitochondrial tethering (Koshiba et al., 2004). While *MFN1* and *MFN2* are distinct, they are still highly structurally related. Each is able to support mitochondrial fusion independent of the other, suggesting partial redundancy in their functions (Chen H. et al., 2003). *MFN1* has displayed higher GTPase activity than *MFN2*, whereas *MFN2* has been specifically implicated in oxidative metabolism, ER-mitochondria tethering, insulin signaling, mitophagy, and apoptosis. *MFN2* gene mutations can lead to autosomal dominant Charcot-Marie Tooth disease, which is discussed in greater detail in the diseases section of this review.

### *Mgm1* and *OPA1*

The proteins *Mgm1* and *OPA1* are DRPs which reside in the mitochondrial inner-membrane and which have exposure to the intermembrane space (Olichon et al., 2002; Wong et al., 2003; Meeusen et al., 2006). *Mgm1*, which is tethered to the inner-membrane by its N-terminal transmembrane domain, is necessary for both outer and inner mitochondrial membrane fusion. Any interruption of the *Mgm1* gene can cause mitochondrial fragmentation and loss of mitochondrial DNA (mtDNA) (Wong et al., 2000; Liang et al., 2018). *OPA1* has also been shown to be crucial for mitochondrial fusion (Alexander et al., 2000; Yarosh et al., 2008). Originally discovered in a study of gene mutation screening of autosomal dominant optic atrophy (Alexander et al., 2000), at least eight mRNA variants are generated *in vivo* as a result of alternative splicing of exons 4, 4b, and 5b. These alternative splices have relatively unknown functions but appear to be functionally important in preserving mtDNA content, energetics, and *cristae* structure, as well as having a role in mitochondrial dynamics. For more information regarding potential functions of these mRNA variants, we direct interested readers to recently published in-depth reviews (Belenguer and Pellegrini, 2013; Del Dotto et al., 2018).

*OPA1* has two distinct isoforms: short and long. The long isoform is generated after *OPA1* is imported into the IMM. The precursor protein contains what is known as a mitochondrial targeting sequence. Cleavage of this sequence generates membrane-anchored long *OPA1* isoforms (*l*-OPA) (Del Dotto et al., 2018). The generation of short isoforms (*s-OPA*) is a result of proteolytic processing of *OPA1* by OMA1 and YME1L, creating a balance between the two (Ali and McStay, 2018). It was initially believed that both *l-OPA* and *s-OPA* were required for mitochondrial fusion (Herlan et al., 2004), but the function of *s-OPA* is a controversial matter. It is generally considered now that the long isoform is sufficient for fusion and that the soluble short isoform may be necessary to facilitate mitochondrial fission (MacVicar and Langer, 2016; Ban et al., 2018). However, recent work still indicates that *s-OPA* plays a role in regulating mitochondrial fusion (Wang et al., 2021).

*Mgm1* also has short and long isoforms. After outer membrane fusion has occurred, the *Mgm1* isoforms come together to form a functional heterodimeric unit that mediates mitochondrial fusion (Zick et al., 2009). It is proposed that the long *Mgm1* isoform is responsible for tethering the opposite inner membrane during fusion whereas the short isoform initiates lipid bilayer mixing in a GTPase-dependent manner (Meeusen et al., 2006; DeVay et al., 2009). It also contributes to local membrane bending, which is mechanically necessary for fusion to occur (Rujiviphat et al., 2015).

While the significance of the different molecular *OPA1* species remains unclear, it is known that both the long and short forms are necessary for full functionality of the protein, as is also the case with *Mgm1*. *Mgm1*/*OPA1* have also been shown to play a role in maintaining the structure of ATP synthase, cytochrome c storage within *cristae*, and *cristae* morphology (Amutha et al., 2004; Del Dotto et al., 2018).

### Ugo1

*Ugo1* is a non-DRP outer-membrane tethered protein containing three transmembrane domains and is specific to yeast belonging to the mitochondrial transporter family. It was initially detected while screening for yeast mutants that lost mtDNA in *Dnm1*-dependent mitochondrial fission (Sesaki and Jensen, 2001). *Ugo1* is proposed to be the bridge between *Fzo1* and *Mgm1*. The N-terminus of *Ugo1* faces the cytosol and the C-terminal domain faces the intermembrane space. Interactions between *Fzo1* and *Mgm1* requires *Ugo1* to be present (Sesaki and Jensen, 2001). Studies have shown that *OPA1* requires *MFN1* to be present for mitochondria to engage in fusion (Alexander et al., 2000; Cipolat et al., 2004; Yarosh et al., 2008). However, as it relates to *Ugo1*, there is no mammalian equivalent that has been identified as a bridge between *OPA1* and *MFN2*. More research is needed to identify the exact mechanism of the proteins’ interactions. Future work also remains to be done to determine the specific mechanisms by which *Ugo1* functions in mitochondrial fusion.

## Mitochondrial fission proteins

There are four key proteins in yeast involved in mitochondrial fission: Dynamin-1 (*Dnm1*), Mitochondrial fission protein 1 (*Fis1*), and adaptor proteins Mdv1 and Caf4.

### *Dnm1* and *DRP1*

*Dnm1* and its mammalian equivalent, *DRP1*, constitute the central components of mitochondrial fission in the majority of eukaryotic organisms. *Dnm1* was originally discovered through the screening of yeast mutants with defective mitochondrial morphology (Hales and Fuller, 1997). *DRP1* contains four identified functional domains. A large N-terminal GTPase domain, a middle domain, the intrinsically disordered variable domain (VD) which is also referred to as the B-insert (Lu et al., 2018), and a C-terminal GED. The middle domain and C-terminal GED form the stalk region consisting of a three-helix bundle known as the bundle signaling element (BSE) that connects to the GED to form a four-helix bundle (Wenger et al., 2013). The VD is a lipid-binding region of approximately 100 amino acids that is found between the third and fourth α-helices of the stalk (Kalia et al., 2018; Kalia and Frost, 2019; Banerjee et al., 2022). The VD a critical component for DRP1 function and regulation. Intramolecular interactions between the GTPase and GED regions are necessary for fission to be fully functional (Zhu et al., 2004). *DRP1* exists mostly as a cytosolic dynamin family member and is recruited to punctate structures on the mitochondrial surface. It then forms spirals to constrict and sever both the outer and inner mitochondrial membranes. *DRP1* and *Dnm1* recruitment is reliant on certain accessory proteins to be successful. These accessory proteins include mitochondrial fission 1 (*Fis1*) and mitochondrial division protein 1 (Mdv1) (Zhang et al., 2012).

### *Fis1, MFF, MiD49* and *MiD51*

**FIGURE 1:**
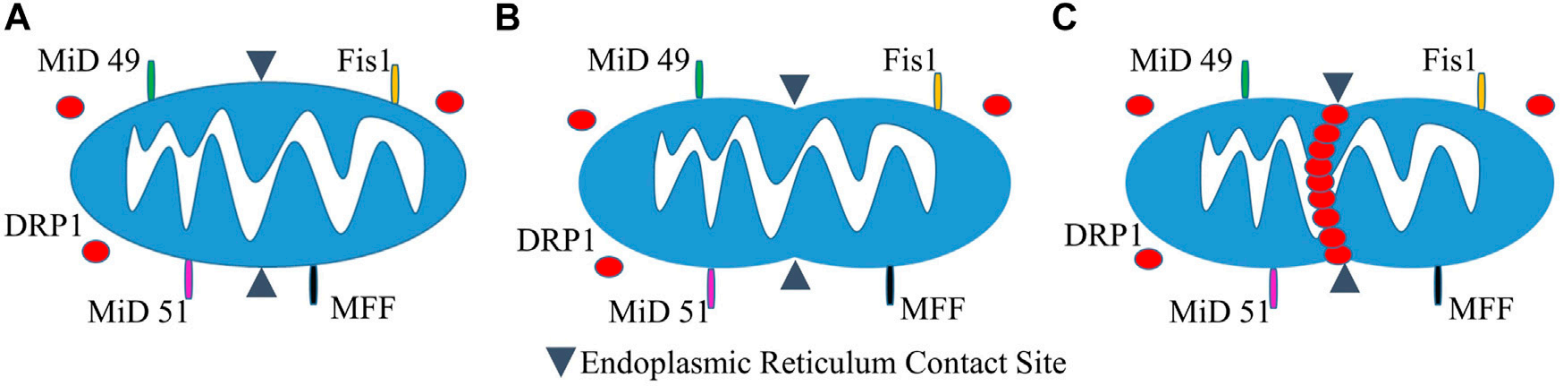
Mitochondrial Fission. **(A)** *Fis1* and potential *DRP1* recruitment candidates MiD 49, MiD 51, and MFF reside on the OMM with most of the protein facing the cytosol. *DRP1* exists in both the cytosol and in punctate spots on mitochondria (not shown). **(B)** An initial constriction of mitochondrial tubules occurs at sites of endoplasmic reticulum contact independent of *DRP1*. **(C)** Mitochondrial fission proceeds as *DRP1* is recruited to the OMM and further constricts the mitochondrial tubule.

*Fis1* is a protein in the outer membrane of mitochondria that recruits *Dnm1* to the surface *via* a molecular adaptor (either Mdv1 or Caf4). Mdv1 acts as a protein bridge between *Fis1* and *Dnm1* by binding its N-terminus to *Fis1* and its C-terminus to *Dnm1* (Zhang et al., 2012). The *Fis1*-Mdv1-*Dnm1* complex is not the only pathway for recruitment of *Dnm1* to mitochondria in yeast. Caf4 is a Mdv1 paralogue with a similar structure. While Mdv1 is present, Caf4 is unnecessary for fission to occur, however, in mutants lacking Mdv1, it acts as a placeholder for fission activity (Griffin et al., 2005). The mechanism and biological importance surrounding Caf4 is still unclear and requires more research. The same can be said for the mechanisms underlying fission in mammalian models (Figure 1). *Fis1* interacts with *DRP1* in a similar way to its yeast ortholog *Dnm1*, as overexpression of *Fis1* leads to the fragmentation of mitochondria and interconnected mitochondrial networks with *Fis1* depletion (James et al., 2003; Yoon et al., 2003). A Mdv1 equivalent has yet to be discovered outside of yeast models, and human *Fis1* knockdown experiments have demonstrated there to be little effect on *DRP1* distribution in mitochondria (Lee et al., 2004). This has led to the assumption that there are alternative pathways for *DRP1* recruitment in metazoans.

Other studies have shown promise for possible candidates for recruiting DRP1, including Mitochondrial Fission Factor (*MFF*) (Gandre-Babbe and Van der bliek, 2008; Otera et al., 2010), and Mitochondrial Dynamics proteins 49 (*MiD49*) and 51 (*MiD51*) (Palmer et al., 2011). The complex interplay between these various species makes the physiological role of *Fis1* in mitochondrial fission, mitophagy, and apoptosis a subject of controversy, given that the dominance and specific roles of *Fis1, MFF, MiD49/51* continue to be clarified. *Fis1* has historically been regarded as a key component of mitochondrial fission due to its role in trafficking *DRP1* to the mitochondria. However, this is being challenged since *Fis1* deletion in some, but not all cell types, is associated with mitochondrial elongation (Ihenacho et al., 2021). This imbalance between fusion and fission has increased interest in the roles of *MFF*, *MiD49* and *MiD51*. *MFF* is currently regarded as the most important regulator of fission as a result of knockdown studies presenting more extreme elongation of mitochondria (Loson et al., 2013). Studies within the last decade of *MFF*’s role in mitochondrial fission indicate its ability to recruit *DRP1* independent of *Fis1* (Loson et al., 2013). In addition, *MiD49* and *MiD51* can act independently of both *Fis1* and *MFF* in trafficking (recruitment of) *DRP1* (Loson et al., 2013). Studies suggest that *MiD49* and *MiD51* interact with *DRP1* and *MFF*, serving as an adaptor to link them together (Palmer et al., 2013; Yu et al., 2017). The data reveal that they work in tandem and that the level of *MiD49/51* creates a balance between mitochondrial fusion and fission. While the particular implications for their mitochondrial dynamics influences remain to be demonstrated, greater detail regarding the roles of *MFF*, *MiD49* and *MiD51* in interactions with fission machinery can be accessed in several excellent publications (Loson et al., 2013; Kraus and Ryan, 2017; Samangouei et al., 2018; Ihenacho et al., 2021).

## Organelle and cytoskeletal interactions

### Endoplasmic reticulum

The endoplasmic reticulum (ER) interacts with mitochondrial membranes at ER contact sites (see Figure 1). These contact sites have been shown to be critical in calcium signaling, phospholipid synthesis, and mitochondrial constriction (i.e., membrane indentation), marking a mitochondrion for fission (de Brito and Scorrano, 2010; Friedman et al., 2011). Initiation of mitochondrial constriction induced by contact with the ER is an essential step toward mitochondrial fission, as DRP1 oligomers alone are unable to induce fission due to mitochondrial size (Friedman et al., 2011). MFN2 has also been found to localize at mitochondrial-ER contact sites where it promotes the fusion of mitochondrial membranes (Koshiba et al., 2004; Abrisch et al., 2020). The emergence of information regarding MFN2 as a crucial regulator of mitochondrial-ER tethering as well as mitophagy has promoted interest in conducting studies regarding mitochondrial diseases and therapeutics, particularly in regard to aging and age-related illness (Moltedo et al., 2019).

### Microtubule interactions

The highly motile nature of mitochondria in mammalian cells is chiefly motivated by molecular motors that actively translocate mitochondria along the microtubule cytoskeleton. In some cell types, approximately 80% of the cell volume is explored by mitochondria within 15 min (Valm et al., 2017). Microtubules are radially organized with their plus ends spreading outward toward the cell periphery, and their less dynamic minus ends clustered at or close to the microtubule-organizing center. Observation of mitochondrial movement in neurons identified proteins, kinesin and dynein, that regulate movement toward these microtubule ends. Dynein and kinesin are in a balancing act with each other and the net directionality of mitochondrial movement resulting from this “tug-of-war” is ultimately determined by the relative number of attached and active dynein and kinesin molecules.

### Dyneins

Cytoplasmic dynein and its activator dynactin, an elaborate multiprotein complex, drive motility inward toward the minus-end of microtubules and act as the primary retrograde motor determined through the same functional analysis techniques as with kinesin-1 (Schnapp and Reese, 1989; Vale, 2003; Pilling et al., 2006), described below. Dyneins are structurally more complex than kinesins and contain a large number of light chains. Dynactin associates with microtubule plus and minus ends and acts as a link to dynein resulting in a dynein-dynactin complex (Schroer, 2004). Additionally, coiled-coil proteins known as activating adaptors are required for the activation of dynein-dynactin motility (Reck-Peterson et al., 2018). Mutations in dynein have been linked to neurological diseases consistent with mitochondrial defects. There is a study however that claims that, in *Drosophila,* dynein heavy chain 64C is the primary motor for both anterograde and retrograde transport of mitochondria (Melkov et al., 2016). This requires further study before any definitive conclusion can be reached.

### Kinesins

Kinesins, which face the synaptic terminal of a nerve cell ending, are the proteins that direct mitochondrial motion toward the plus-ends of axons (Hirokawa et al., 2009). Kinesin-1 was identified as the primary anterograde motor for long-distance intracellular trafficking according to functional inhibition and biochemical studies (Hurd and Saxton, 1996; Pilling et al., 2006). These studies, however, also showed a significant reduction in retrograde flux, controlled by cytoplasmic dynein, in the mutant *Drosophila*. This suggests a possible functional interdependence between mitochondria-associated dynein and kinesin-1. Since the kinesin-1 mutant showed no signs of mitochondrial fission in the axons, dynein-dynein retrograde transport does not occur because kinesin-1 is necessary for mitochondria to be carried into the axon (Pilling et al., 2006). This theory is backed up by showing a 50.5% decrease in the number of mitochondria in the mutant nerves. It should be noted as well that in tissue culture specifically, there are studies that have shown that kinesin-3 motors Klp6 and Kif1b also transport mitochondria, leading researchers to conclude that it also does so in axons (Nangaku et al., 1994; Tanaka et al., 2011).

### Milton and Miro

The best-studied and understood adaptors for associating kinesin-1 with mitochondria are Milton and Miro. Kinesin-1 lies within the kinesin heavy chain (KHC) and interacts with Milton, a kinesin-binding protein crucial for the mediation of mitochondrial motility discovered through genetic screening for *Drosophila* mutants with impaired neuronal function (Stowers et al., 2002). Milton binds directly to the C-terminal cargo-binding domain of the KHC. Studies also indicate that a mutated Milton isoform inhibits retrograde mitochondrial transport from nurse cells into future oocytes during oogenesis in *Drosophila* (Cox and Spradling, 2006). This shows that Milton may exert an effect on dynein specific to that isoform.

The mammalian homologs, trafficking kinesin proteins 1 (*TRAK1*) and 2 (*TRAK2*), similarly engage with kinesin-1 and dynein/dynactin to induce mitochondrial motility. We have demonstrated that *TRAK1* plays an important role in mitochondrial trafficking in a study that identified deleterious variants in *TRAK1* from encephalopathic patients (Barel et al., 2017). *TRAK1*-deficient fibroblasts from this study displayed unorthodox mitochondrial distribution, diminished membrane potential, modified mitochondrial motility, and reduced mitochondrial respiration. *TRAK2* has recently been demonstrated to form a complex containing both kinesin and dynein-dynactin through co-immunoprecipitation and colocalization experiments. Thus it is thought that *TRAK2* acts as an interdependent motor complex that provides integrated control over the two opposing motors (Fenton et al., 2021).

Miro is a mitochondrial Rho-like GTPase containing calcium binding motifs. It is an integral mitochondrial outer membrane protein that interacts with the scaffolding proteins Milton/TRAKs 1 and 2 (Fransson et al., 2003; Guo et al., 2005). Unique to other Rho GTPases, Miro-1 and Miro-2 have been shown to have roles in mitochondrial homeostasis, most likely affecting the trafficking apparatus (Vale, 2003). Miro may be preferentially needed for retrograde mitochondrial transport (Melkov et al., 2016). Mutation of either Milton or Miro genes halts any anterograde mitochondrial axon transport, depleting mitochondria in the axon and synapse leading to an aggregation of mitochondria and eventual apoptosis. Miro has also been found to be required for mitochondrial transport from axons to synapses in *Drosophila* (Guo et al., 2005). Mutant flies lacking Miro display mislocalized mitochondria in both muscles and neurons as well as a lack of presynaptic mitochondria in neuromuscular junctions. These findings suggest that Miro plays an important role in transporting mitochondria to nerve ends.

Other adaptor complexes that have been associated with a linkage between kinesin and mitochondria include kinectin, an integral endoplasmic reticulum (ER) membrane protein that targets kinesin to the ER membrane (Santama et al., 2004), and syntabulin, which contains a syntaxin-independent mitochondrial binding site. When syntabulin is inhibited, anterograde mitochondrial movement is disrupted (Cai et al., 2005).

### Syntaphilin

Syntaphilin (Snph) anchors mitochondria to microtubules, rendering them stationary. Snph behaves as an engine off-switch by detecting Miro-Ca^2+^ and it acts as a brake by tethering mitochondria to the microtubule track (Ohno et al., 2014). Snph facilitates the survival of demyelinated axons by increasing the volume of static mitochondria. The deletion of axonal Snph significantly increases the deterioration of demyelinated central nervous system axons indicating a need for mitochondrial stationary sites to meet the increased energy demands of an unmyelinated axon (Ohno et al., 2014).

### Intermediate filaments

Links between intermediate filaments (IFs) and mitochondria appear in the colocalization of mitochondria with neuronal IFs (Wagner et al., 2003). There is also evidence that IFs are involved in mitochondrial tethering. IFs interact with mitochondria by surrounding the organelle by confining or binding the mitochondrial membrane *via* a protein known as plectin (Schwarz and Leube, 2016). Mutations in IF proteins result in the disruption of IF networks. The deletion of desmin, an IF protein, alters the morphology, distribution, and respiratory function of mitochondria in striated muscle cells (Milner et al., 2000). In addition, desmin deletion inhibits kinesin-1 recruitment to mitochondria in heart muscle (Linden et al., 2001). Vimentin IFs (VimIFs) affect the transportation and tethering of mitochondria. Knock-Out of VimIFs in fibroblasts showed altered mitochondrial distribution, and increased motility (Nekrasova et al., 2011). The modulation of mitochondrial motility due to VimIFs in cultured cells is directly related to its interactions between mitochondria and cytoskeletal structures. More research needs to be done regarding the regulation between IFs and mitochondria. As of now there is no conclusive evidence for IF-associated motor molecules and IFs might have more influence over microtubule motors than is in the literature.

## Actin interactions

A 1995 study was the first to show that mitochondria directly interact with the actin cytoskeleton, and that there is an implication in motility (Simon et al., 1995). The actin cytoskeleton guides mitochondrial translocation in simple eukaryotes such as budding yeast and plays pivotal and diverse roles mediating the mitochondrial network and function. While long-range mitochondrial motility is directed by microtubules, the actin cytoskeleton specializes in coordinated short range mitochondrial motion (Quintero et al., 2009) and anchoring (Pathak et al., 2010). This is carried out by myosins, which are a family of actin-dependent motor molecules.

### Myosin

Myosin is a motor protein known mostly for its role in muscle contraction and is ATP-dependent and responsible for actin-based motility. Myosin directs movement toward actin filament plus-ends, such as myosin V (Altmann et al., 2008), or minus-ends like myosin VI (Lister et al., 2004). There are also some indications that Myo2 may be involved in mitochondrial motility more than indirectly (Altmann et al., 2008). While there is a large number of actin-mitochondria interactions, there lacks critical information as to how mitochondria are linked to the filamentous actin (F-actin) cytoskeleton. The best evidence for actin-based mitochondrial motility comes from the myosin motor Myosin XIX (Myo19). Similar to myosin V, it is a plus-end directed motor but it is known to localize specifically to the OMM to drive mitochondrial motility (Lu et al., 2014). While it is an example of an actin binding protein interacting directly with the OMM, its occurrence is limited to the starvation induced filopodia in select cell lines, and does not provide any further information as to how mitochondria are tied to actin in other situations. Actin-based mitochondrial motility is still under much scrutiny as the mechanism by which it affects motility is ambiguous. Studies provide information on opposing motor dynamics without information as to how it occurs. Myosin V is said to promote motility in one study (Hollenbeck, 1996) while F-actin and Myosin V are said to be resistors of mitochondrial motility (Pathak et al., 2010; Gutnick et al., 2019).

## Evidence for mitochondrial dynamics

Advances in imaging techniques and technology have enabled mitochondrial fusion and fission events to be quantitatively ascertained and visually observed. The best indirect methods for the assessment of mitochondrial fusion and fission in mammalian cells include cell-cell fusion, photoactivatable mitochondrially target Green Fluorescence Protein (mitoGFP), and fluorescence recovery after photobleaching (FRAP). Cell-cell fusion is a technique used to observe the mixing of mitochondrial matrices in mammalian cells. It was experimentally induced between two cell populations labeled with two differing mitochondrial markers, either mitoGFP or mitochondrially targeted Red Fluorescence Protein (Legros et al., 2002; Malka et al., 2005).

FRAP was developed in order to observe mitochondria under in vivo-like conditions by avoiding potential artifacts from membrane-altering agents. FRAP is performed by photobleaching fluorescent molecules within a subcellular area and monitoring its recovery of fluorescence in the bleached zone by observing the movement of organellar structures, in this case mitochondria, from an unbleached area. Information acquired from such experiments includes the mobility of the fluorescent molecule, mitochondrial continuity, and mitochondrial dynamics (e.g., motility, fusion, fission). Mitochondrial connectivity is confirmed with rapid recovery of fluorescence while a failure of recovery is an indication of mitochondrial fragmentation or discontinuity (Collins et al., 2002). An example of this approach is its use in determining that CED-9, a *C. elegans* Bcl-2 homolog, bolsters mitochondrial fusion in HeLa cells (Delivani et al., 2006).

Use of photoactivatable fluorophores such as mitoGFP is widely used to assess mitochondrial fusion and fission events. Photoactivatable mitoGFP is designed to display a 100-fold increase in green fluorescence after laser activation (Patterson and Lippincott-Schwartz, 2002) and can be fused to a mitochondrial targeting sequence. This approach was used in a study that showed Bcl-2 family proteins *BAX* and *BCL2* antagonist/killer (*BAK*) to have direct roles in mitochondrial fusion (Karbowski et al., 2006). An observation of a loss of photoactivated fluorescence in an area can be indicative of mitochondrial fusion events if one assumes that this loss is a representation of fluorescent diffusion into a neighboring mitochondria, while the opposite can be said for mitochondrial fission. The best markers are considered to be mitoGFP, mitoRFP, and mitochondrially targeted Yellow Fluorescence Protein (mitoYFP), as they have excitation peaks at a longer wavelength to avoid possible mitochondrial damage that a more energetic and shorter wavelength would provide during recording of a time-lapse microscopy-based assessment.

A more direct method uses photoconvertible fluorophores, which differ from photoactivatable fluorophores by emitting fluorescence in their non-converted state. Dendra2 was the first monomeric red-to-green photoconvertible protein to be commercially developed, and it was used to create the mito-Dendra2 mouse model. The mito-Dendra2 mouse model has been extensively used to track mitochondrial fusion and fission *in vivo* and *ex vivo* in skeletal muscle fiber (Mishra et al., 2015), cerebral vasculature (Rutkai et al., 2020), and hematopoietic stem cells (Takihara et al., 2019), among many other cells and tissues. The mito-Dendra2 mouse has also been used to display mitochondrial transfer activities from early stage erythroblasts to macrophages *in vitro* (Yang et al., 2021), and axonal mitochondrial dynamics in regenerative response to injured neurons (Han et al., 2016).

## Characteristics of mitochondrial motility

The ability to quantify mitochondrial motility has been largely driven by advances in live cell imaging techniques and image processing. A number of fluorophores have been developed which are specific for mitochondria or which accumulate in mitochondria based on membrane potential. These dyes can be used to localize mitochondria within cells. When coupled with particle tracking-based image analysis of time-sequentially generated microscopy frames, motion of individual mitochondria can be determined. Kandel et al. (Kandel et al., 2015) utilized this approach and were able to demonstrate that a population of hundreds of mitochondria tracked simultaneously followed a log-normal distribution in their net displacement over time. These investigators found that motility was dependent on cytoskeletal structure, with motility increasing in the presence of depolymerized actin and motility decreasing with inhibition of microtubule production. Further work has demonstrated alterations in mitochondrial motility in cells undergoing various forms of environmental or chemical stress such as rapid decompression from hyperbaric conditions (Jang et al., 2018) and carbon monoxide poisoning (Owiredu et al., 2020) as well as critical illness (Jang et al., 2019). Both molecular and environmental therapies have been shown to have capabilities to restore disturbed motility to baseline values (Ranganathan et al., 2020; Green et al., 2021). These findings demonstrate that motility characterization is potentially a useful clinical index of mitochondrial dynamics dysfunction and this raises the possibility of using motility assessment for surveillance of disease progression or effectiveness of therapeutic intervention.

## Methods to assess mitochondrial motility

Assessment of mitochondrial motility relies primarily on imaging-based methods to track individual mitochondrial movements. Mitochondrial tracking is technically challenging owing to the small size and complexity of their movements. Tracking individual mitochondrial motion manually is time-consuming and extremely prone to errors. Most studies have traditionally focused on the tracking of neuronal mitochondria because they are more sparse and hence can be individually well-resolved, making them most suitable to be tracked. Some methods for ascertaining mitochondrial motility in generalized cell types are described in a paper from De Vos and Sheetz (De Vos and Sheetz, 2007). The first method relies on use of two consecutive images of a cell from which the non-overlapping region stained with a fluorescent dye is measured as a whole-cell index of mitochondrial motility. Another method utilizes a kymograph, which evaluates space *versus* time images for individual mitochondria.

Confocal microscopy (Koopman et al., 2006; Bros et al., 2015) and time-lapse fluorescence microscopy imaging (Kandel et al., 2015; Chalmers et al., 2016) are the most commonly used methods to image cells that are stained with a fluorescent dye specific to mitochondria. Successive images are acquired over varying amounts of time depending on the specific methodology being used. In a majority of the techniques, the sequence of images is made into a stack that is processed by a third-party program or algorithm for image analysis. Resultant visualizations of individual mitochondria can be observed and specific measurements of mitochondrial displacements can be determined.

For purposes of analyzing mitochondrial motility through image processing, there are a few noteworthy methods. The Mitochondrial Network Analysis (MiNA) (Valente et al., 2017), which can be accessed at https://github.com/StuartLab/MiNA, utilizes the freeware program ImageJ to quantitatively describe the morphology of mitochondria present in fluorescence micrographs. This software is optimized for 2D images, but it has some limited functionality for 3D, especially if it is used for simple visualization purposes. MiNA does not enable any temporal analysis to be performed since it only outputs cell-wide aggregate mitochondrial data.

Mitograph (Viana et al., 2015), accessible at https://github.com/vianamp/MitoGraph, is a program that is fully automated for analyzing the 3D morphology, volume and topology of mitochondria in living cells. This software has been optimized and validated for tubular mitochondrial networks in budding yeast (Viana et al., 2020), which may limit its use for other applications. With additional validation it may be applied to the study of other cell types. Only a single temporal frame can be viewed with this package, thus restricting the use of this imaging method for making any measurements of motility. The program runs exclusively through command lines and is a free software written in C++; it does not have a graphical user interface (GUI).

Mitoe (Lihavainen et al., 2012), a freely available resource, is online, located at https://sites.google.com/view/andreribeirolab/home/software,. It can be used to analyze mitochondrial dynamics and structure from 2D fluorescence microscopy images. It is not clear how a temporal sequence of images is handled by the optical flow algorithm, which analyzes two adjacent frames at a time but apparently does not record displacement tracks established between frames. Also, multiple image stacks cannot be analyzed simultaneously with this software, as only a single frame from each temporal stack of images gets processed. Individual analysis of stacks must be performed first and then they can subsequently be manually aggregated. The program is written in MATLAB, however a standalone Windows version that does not require MATLAB is available. One user challenge with this software is that the MATLAB GUI is not fully operational.

**FIGURE 2:**
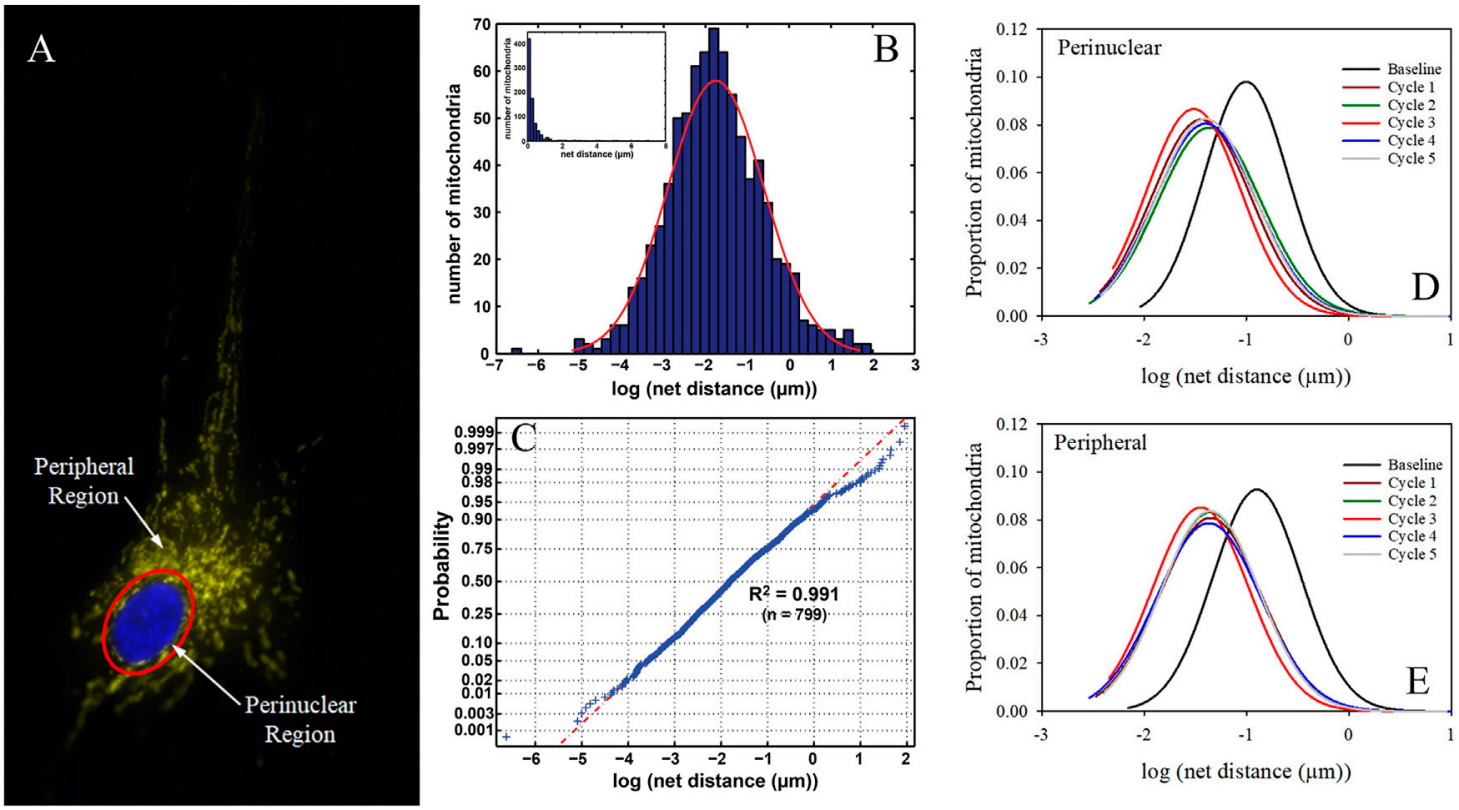
Intracellular partitioning of mitochondria and lognormal distribution of mitochondrial motility. **(A)** The image of a fibroblast shows a nucleus stained with DAPI (blue) and TMRM-stained mitochondria (yellow). The mitochondria are separated into peripheral and perinuclear regions by the image-analysis derived partition (red). **(B)** Example of distribution of net distances traversed by hundreds of mitochondria on a log scale, with inset showing linear scaling of the same data (Kandel et al., 2015). **(C)** Normal probability distribution of the log values of net mitochondrial distances traversed indicating that motility follows a lognormal distribution (Kandel et al., 2015). **(D)** Lognormal distribution of mitochondrial motility in cellular perinuclear region at baseline and following multiple hyperbaric oxygen exposures as detailed in (Green et al., 2021). **(E)** Lognormal distribution of mitochondrial motility in cellular peripheral region at baseline and following multiple hyperbaric oxygen exposures as detailed in (Green et al., 2021).

Our own Mitochondrial Single Particle Tracking (MitoSPT) (Kandel et al., 2015), is available for download at https://github.com/kandelj/MitoSPT. This program is written in MATLAB and has a user-friendly GUI. The algorithm, which is clearly annotated, utilizes analysis of 2D imaging to provide a depiction of mitochondrial motility, rates of fusion and fission, morphometric parameters (mitochondrial number, size) and statistical analysis for comparison between image stacks obtained from individual experimental conditions. In addition, the software has provision for subdivision of the measured parameters to be analyzed in two distinct intracellular regions, a thin perinuclear region and the remaining cell periphery, as depicted in Figure 2, rather than on a whole-cell basis (Jang et al., 2019; Green et al., 2021). This software was instrumental in the initial determination that mitochondrial motility follows a lognormal distribution as seen in Figure 2 (Kandel et al., 2015) and in demonstrating that motility in the cell perinuclear and peripheral regions is not identical, as is shown in Figure 2 (Jang et al., 2019; Green et al., 2021).

QuoVadoPro (QVP) (Basu and Schwarz, 2020) is also available online and can be found at https://github.com/ThomasSchwarzLab/QuoVadoPro, utilizes ImageJ macros to quantify movement of a fluorescently tagged organelle. The extent of intracellular movement is inferred by quantifying the variance of pixel illumination over a series of time-lapse images. QVP gives only a motility score as a motility metric on an image-wide basis and not by frame-by-frame quantification. Mitochondrial morphometrics are not analyzed nor are fission and fusion events. QVP is implemented as an ImageJ plugin.

Mitochondrial Segmentation Network (MitoSegNet) (Fischer et al., 2020), found at https://github.com/MitoSegNet, uses a pre trained deep learning model that segments mitochondria and analyzes mitochondrial morphometrics. There is no need for user-input parameters with this software, which handles batch input and thus eliminates bias. MitoSegNet analyzes 2D images from fluorescence microscopy but does not perform analysis of temporal variation. It can be used only to visualize and quantify individual mitochondrial morphometrics. This program is based in Python and runs on a standalone software platform.

MTrack2, found at https://github.com/fiji/MTrack2, utilizes ImageJ’s particle analyzer plugin to link particles between frames of 2D images. This is a fairly simple 2D tracking software package that gives centroid positions of objects in a binary image and tracks them over time. No morphology or fission and fusion events can be analyzed through this software. MTrack2 is an ImageJ plugin written in Java, and its GUI is relatively limited because it relies on ImageJ’s templates and results page to provide results output.

## Regulation of mitochondrial morphology

Mitochondria form tubular networks that undergo changes in morphology to correspond with cellular needs through fusion and fission events. The shape of mitochondria can have an effect on their distribution to specific subcellular locations. This has been found to be especially important in neurons, in which small mitochondria are more efficiently transported to nerve terminals. It is however worth noting that smaller mitochondria do not always engage in efficient transport and that fragmented mitochondria resulting from a fusion impairment show signs of critical transport defects (Chen H. C. et al., 2003). Mitochondrial size and shape may also be directly related to bioenergetic function as elongated mitochondria have been shown to be correlated with more effective ATP production. The distinct connection between the shape of mitochondria and bioenergetic function still remains unclear.

Mitochondria are known to be responsive to cues that change their morphology. Under conditions of starvation and stress, stress-induced mitochondrial hyperfusion (SIHM) is triggered. During SIHM episodes, mitochondria will hyperfuse into large complexes to offer cytoprotection and enhanced resistance to apoptosis, increasing opportunity for the cell to return to homeostasis (Tondera et al., 2009). Apoptosis activation, oxidative stress, and cell senescence have also been shown to induce mitochondrial elongation. The elongation of mitochondria during the G1-S phase might play a role as a mediator in stress-induced premature senescence (Yoon et al., 2006). Nutrient excess on the other hand leads to mitochondrial network fragmentation, an apparent response to the luxury of available bioenergetics substrate (Liesa and Shirihai, 2013).

## Content exchange in mitochondria

A primary function of mitochondrial fission and fusion is the promotion of the membrane and contents mixing between mitochondria. Kiss-and-run fusion events, or transient fusion, progress content exchange without having an effect on mitochondrial morphology (Liu et al., 2009). Labeled mitochondria have been shown to exchange matrix contents during these transient fusion events, which constitute nearly half of all fusion events resulting in the exchange of matrix proteins between mitochondria. This suggests that fusion pores between two mitochondria can open and close rapidly and furthers the suggestion that aside from the regulation of morphology, fusion plays a large role in content exchange between mitochondria.

The benefits of content exchange include the promotion of homogenization of the mitochondrial population as well as amelioration of injurious effects of heteroplasmic mtDNA mutations. Since the mtDNA only encodes for 13 polypeptides, most of the mitochondria’s proteasome is imported from the cytosol. The inhibition of mitochondrial fusion causes individual mitochondria to deviate in their properties, indicating that fusion assists in the reduction of variability between organelles. As most mtDNA mutations are recessive in nature, the mutational load must attain high levels of around 60–90% heteroplasmy before any appreciable dysfunction in the respiratory chain is encountered. The capacity to withstand these excessive levels of mtDNA mutations is extremely reliant on mitochondrial fusion (Chen et al., 2010).

## Mitochondrial inheritance

Mitochondria cannot be created *de novo*; thus, they must be inherited by daughter cells through mitosis. Elongation of mitochondria occurs during the G1-S phase in tissue culture cells but then fragmentation begins at the G2 and M phases (Mitra et al., 2009). Mitotic phosphorylation of *DRP1* promotes this fragmentation that enhances the rate of fission and results in the production of many more mitochondria during mitosis (Taguchi et al., 2007). This is important because mitochondria have affiliations with elements in the cytoskeleton that are dispersed throughout the cell (see above), and this fragmentation might assist with the even dispersal of mitochondria between the two daughter cells.

Mitochondrial morphology changes that are cell-cycle dependent are not as noticeable in yeast because their mitochondria are inherited in an organized manner by cytoskeleton-dependent bud-directed transport. A lack of *Dnm1* in yeast mutants does not show a notable growth defect despite having dramatic morphological changes, even though, during cell division, their interconnected mitochondrial network must undergo division (Sesaki et al., 2014). As division still occurs, the mitochondria sustain no loss in functionality. Additionally, the knockout of *DRP1* in mouse embryonic fibroblasts does not affect cytokinesis progression, however this results in embryonic lethality in mice (Ishihara et al., 2009). It is unclear whether the division of mitochondria in mutated cells is regulated by mechanical forces that act on the mitochondria during cytokinesis or by an unknown division machinery. Since mice lacking *DRP1* die before they are birthed, there is an indication that mitochondrial dynamics are critical for development and survival (Song et al., 2017).

Disruption of fusion in yeast is associated with a rapid loss of mitochondrial genome and resultant defects in respiration. This is most likely due to fragmentation of mitochondria producing multiple mitochondrial fragments with many of them lacking mtDNA. The progeny produced from the partitioning of these mitochondria to daughter cells results in mitochondrial genomes being absent from the population after a few generations. Mitochondrial fusion makes certain that the loss of gene products and the mitochondrial genome lost due to fission are replaced before full functionality is impaired.

## Mitochondrial DNA maintenance

Mitochondrial fission and fusion are both essential in the maintenance of mtDNA. With the loss of mitochondrial fusion through the silencing or removal of necessary proteins, cells display an extreme reduction in the amount of mtDNA they contain. Many of the mitochondria in mutant mammalian cells lack evidence of any mtDNA, which renders them unable to maintain oxidative phosphorylation (OXPHOS) activity (Chen et al., 2007). The loss of mitochondrial fusion in many cell types reduces OXPHOS activity, however it is unclear whether this defect is a product of the reduced mtDNA levels or if an additional mechanism akin to fusion is involved.

When mitochondrial fission is absent, the mtDNA nucleoids begin to aggregate and form large structures that distort mitochondrial tubules (Ban-Ishihara et al., 2013). This aggregation causes an unbalanced dispersion of mtDNA in the mitochondria, with studies using cardiomyocytes having shown that this results in mosaic OXPHOS deficits that promote cardiac arrhythmia during aging (Ishihara et al., 2015).

## Mitophagy and apoptosis

Mitochondrial fission acts as a regulator, identifying poorly functioning organelles that display reduced mitochondrial membrane potential (Twig et al., 2008). Mitochondria may recover as discussed above through fusion and fission. Alternatively, mitochondria may be targeted for mitophagy, which is the degeneration of mitochondria by autophagy (Nguyen et al., 2016; Dikic and Elazar, 2018). Mitophagy is an adaptive survival mechanism that prevents the cell from undergoing apoptosis and allows the cell to maintain a healthy pool of mitochondria for sustained energy production. Autophagic machinery within the cell recognizes dysfunctional mitochondria, which become engulfed by autophagosomes and are subsequently delivered to lysosomes for degradation. There has been important recent work clarifying the relationship between mitochondrial fission and mitophagy. While *DRP1* and mitochondrial fission have previously been thought to be necessary for mitophagy to occur in mammalian cells, some recent research findings demonstrate that certain forms of mitophagy do in fact occur independent of *DRP1* (Murakawa et al., 2015; Yamashita et al., 2016). Another study has revealed that the loss of *DRP1* actually enhances mitophagy *in vitro* (Burman et al., 2017). This form of mitochondrial quality control actively functions in both pathological and physiological conditions as a protector from damage and stress inflicted beyond the protein level. Dysfunctional mitochondria which are beyond a level amenable to mitochondrial dynamics repair must be culled from the mitochondrial population. Mitophagy is likely to have a functional association with mitochondrial dynamics as interactions between mitochondrial dynamics factors and LC3 adapters, specifically optineurin, become more apparent (Moore and Holzbaur, 2016). The *PINK1*/*Parkin* pathway, while better known for being genetic factors of Parkinson’s disease, is now known to also play a role in mitophagy (Nguyen et al., 2016; Yoo and Jung, 2018). The biomolecular specifics of the association between mitophagy and mitochondrial dynamics remain to be determined.

Mitochondrial fragmentation through fission has also been well observed in cells undergoing apoptosis, a form of programmed cell death that is critical for development and for adult tissue homeostasis in all multicellular organisms. A key event during the apoptotic process is mitochondrial outer membrane permeabilization (MOMP). As a result of MOMP, there is a release of pro-apoptotic factors including cytochrome c from the intermembrane space to enter into the cytosol, and this triggers downstream cell death pathways. *BAX* is responsible for executing this event (Brady and Gil-Gomez, 1998). *BAX* is recruited to the mitochondrial outer membrane after activation in the cytosol, which leads to oligomer formation and MOMP. This brings the cell to the ‘point of no return’ in the apoptotic pathway. *BAK* has been observed interacting with *DRP1* and mitofusins. This indicates that there does exist crosstalk between the separate machineries of mitochondrial dynamics and apoptosis. While experimental results have shown that the *BAX* oligomerization is promoted by the membrane tethering activity of *DRP1*, *DRP1* itself is not necessary for the initial recruitment of *BAX* to the outer mitochondrial membrane (OMM) (Jenner et al., 2022). This points toward an initiation of MOMP by *BAX* that is independent of the function of *DRP1*, however, *DRP1* promotes the further aggregation of *BAX* in later phases of recruitment. Additional studies are needed to determine exactly how mitochondrial fusion and fission components actively participate in apoptosis.

## Regulation of mitochondrial fusion and fission

### Fusion

The fusion and fission proteins discussed in the previous section are targets for a number of post-translational modifications (PTMs). PTMs facilitate quick responses to alterations in physiological demands and are common mechanisms to modify protein activity. The most studied modifications include acetylation, phosphorylation, ubiquitination, O-GlcNAcylation, and SUMOylation. Mitochondrial fusion is the result of adjacent mitochondria tethering to one another followed by the fusion of the outer mitochondrial membrane (OMM) and the inner mitochondrial membrane (IMM), in that order (Mattie et al., 2019). The regulation of mitochondrial fusion events is carried out through transcription and post-transcriptional and post-translational modifications.

The fusion of adjacent mitochondria is under the regulation of Mitofusins 1 and 2 as discussed previously in this article. A study done in 2006 in Barcelona showed upregulation of *MFN2* expression in mice due to an increased demand for energy by the protein peroxisome proliferator-activated receptor gamma coactivator 1-alpha (PGC-1a), such as skeletal muscle due to increased mechanical load/exercise or brown adipose tissue reacting to cold environments (Soriano et al., 2006). It has also been demonstrated *in vitro* that PGC-1a stimulates the activity of *MFN2* through an interaction with estrogen related receptor-alpha (ERR alpha) (Cartoni et al., 2005). Another disruptor of mitochondrial fusion is seen during the excitotoxicity of neuronal mitochondria. The transcription factor MEF2 has been shown to regulate basal expression of *MFN2* in neurons and during excitotoxicity, MEF2 is degraded resulting in neuronal mitochondria fragmentation and a downregulation of *MFN2* is seen (Martorell-Riera et al., 2014).

Some notable miRNAs have been shown to reduce the expression of *MFN2*, such as mi2R-761 in hepatoma cells (Zhou et al., 2016), miR-106B in breast cancer cells (Wu et al., 2016), and miR-214 in neuroblastoma cells (Bucha et al., 2015). Post-translational modifications that influence mitochondrial fusion include the ubiquitination of *MFN2* by E3-ubiquiting ligases, marking *MFN2* for proteasomal degradation and an overall inhibition of mitochondrial fusion. These include PARKIN (Gegg et al., 2010), MARCH5 (Nakamura et al., 2006), HUWE1 (Leboucher et al., 2012), and MUL1 (Peng et al., 2016a). In the cellular response to stress signals, *MFN2* can also be phosphorylated by *PINK1* and JNK (Leboucher et al., 2012; Chen et al., 2013). They are subsequently marked for ubiquitination by PARKIN and HUWE1 respectively.

MiRNAs are also at the forefront of regulating *MFN1* expression. MiR-140 is known to increase in response to genotoxic or oxidative stress, negatively regulating *MFN1* translation in cardiomyocytes (Li J. et al., 2014). This of course induces mitochondrial fragmentation. *MFN1* has also been found to be downregulated in osteosarcoma cells by miR-19b (Li X. et al., 2014). PARKIN is also involved in the ubiquitination of *MFN1* (Gegg et al., 2010). Post-translational phosphorylation by extracellular-signal-regulated kinase (ERK) also leads to a decrease in mitochondrial fusion (Pyakurel et al., 2015).

The upregulation of mitochondrial fusion is a common response to cellular stress (Tondera et al., 2009; Rambold et al., 2011), with oxidative processes serving as a common pathway for cellular stress to occur. Oxidized glutathione is known to be a primary cellular stress indicator. Elevation of the intracellular level of oxidized glutathione has been shown to induce mitochondrial fusion by promoting the activity of mitofusins (Shutt et al., 2012; Mattie et al., 2018). Results of *in vivo* experiments have demonstrated that exposure to sublethal or low doses of hydrogen peroxide can lead to mitochondrial hyperfusion occurring (Yoon et al., 2006; Shutt et al., 2012). Additionally, there is evidence that some minimum level of ROS or oxidation may be required for the activation of fusion to occur (Shutt et al., 2012).

Other biomolecules play important roles in the regulation of mitofusion activity. The soluble form of BAX, the proapoptotic protein discussed previously, localizes *MFN2* to fusion sites and is a positive regulator of mitochondrial fusion (Hoppins et al., 2011). *In vitro* studies indicate that Bcl2 proteins may play important mitochondrial dynamics housekeeping roles (Karbowski et al., 2006). Mitochondrial carrier homologue 2 (MTCH2) is known as a regulator of mitochondrial metabolism and apoptosis as it is a repressor of mitochondrial OXPHOS (Yuan et al., 2021). However, *in vitro* studies involving embryonic stem cells (ESCs) show that MTCH2 may serve as a novel regulator of mitochondrial fusion, because knockout of MTCH2 from ESCs results in failure of mitochondria to elongate (Bahat et al., 2018). A link between mitochondrial fusion and lipogenesis by way of MTCH2 has also been reported (Labbe et al., 2021), however to date, just how MTCH2 regulates mitochondrial fusion and morphology has not been resolved.

As discussed previously, *OPA1* is in charge of fusion of the IMM. In cardiomyocytes, the transcription factor NF-kB regulates *OPA1* in response to insulin by the way of the Akt-mTOR signaling pathway (Parra et al., 2014). IMM fusion and morphology relies on maintaining the balance between the long and short *OPA1* isoforms. Post-transcriptional and post-translational regulation plays a role in the cleavage of *OPA1* to produce these isoforms. Mitochondrial proteases m-AAA (OMA1) and YME-like protein 1 (YME1L) have been shown to cleave *OPA1* (Anand et al., 2014). Proteolytic processing of OPA1 by YME1L *in vitro* has been found to be regulated by the level of OXPHOS (Mishra et al., 2014). Under high levels of OXPHOS, OPA1 is more efficiently cleaved.

### Fission

Translation, signaling molecules and post-translational modifications promote fragmentation of mitochondrial networks and accelerate mitochondrial fission. The main protein involved in mitochondrial fission regulation is *DRP1* as discussed previously. The miRNA miR-30 helps to activate *DRP1* expression during apoptosis as decreasing levels of miR-30 cause an upregulation of p53, a well-known tumor suppressor protein that transcriptionally activates *DRP1* (Li et al., 2010). Post-translational modifications that play a role in *DRP1* activity and translocation to mitochondria include phosphorylation, ubiquitination, SUMOylation, nitrosylation, and O-GlcNAcylation. Serine 616 (SER616) in mice and Serine 637 (SER637) in human *DRP1* are the most well studied sites of regulation. SER616 is phosphorylated by cdk1, PKC, ERK1-2, and CaMKII resulting in *DRP1*-induced mitochondrial fission. On the other hand, this process is inhibited by PKA phosphorylation of SER637 (Chang and Blackstone, 2007; Cribbs and Strack, 2007; Santel and Frank, 2008; Kashatus et al., 2015; Serasinghe et al., 2015). SER637 can also be dephosphorylated by calcineurin and produces an opposite effect as the blockage of calcineurin results in a prevention of translocation (Cribbs and Strack, 2007; Cereghetti et al., 2008; Kim et al., 2011). It has also been shown that miR-499 is involved in repressing calcineurin and inhibiting pro-fission activity (Cereghetti et al., 2008). Downregulation of calcineurin by miR-499 has been shown to provide protection from mitochondrial fragmentation in cardiomyocytes in response to ischemia-reperfusion (Wang et al., 2011). Other kinases involved in the phosphorylation of *DRP1* that result in a decrease of *DRP1* levels and translocation to mitochondria include Pim-1 at SER637 (Din et al., 2013) and GSK3b at SER693 (Chou et al., 2012). Kinases that resulted in an increase in *DRP1* and the promotion of mitochondrial fission include ROCK1 (Wang et al., 2012) and CaMKIa (Han et al., 2008), both at SER637, as components of the response to hyperglycemia or increased levels of calcium.

SUMOylation plays an important role in regulating *DRP1* protein stability. MAPL, a mitochondria-anchored SUMO E3-ligase, SUMOylates *DRP1* resulting in mitochondrial fission (Braschi et al., 2009). SENp5, a SUMO protease, alternatively deSUMOylates *DRP1* by removing SUMO-1 from *DRP1* inhibiting mitochondrial fission (Zunino et al., 2007). PARKIN and MARCH5 are also involved in fission processes by mediating the ubiquitination of *DRP1*. In neurons, PARKIN has been shown to degrade *DRP1* and suppress mitochondrial fission (Lutz et al., 2009). The opposite occurs with MARCH5 as it regulates subcellular trafficking of *DRP1* leading to an increase in mitochondrial fission (Karbowski et al., 2007). The most likely explanation is that it affects the disassembly of fission complexes or the precise assembly at scission sites. O-GlcNAcylation of *DRP1* in cardiomyocytes has also been shown to have an effect on mitochondrial fission. The first study to display this showed that during high glucose treatment of N-acetyl-glucosaminidase inhibition, Threonine 585 (THR585) and Threonine 586 (THR586) are O-GlcNAcylated leading to a reduction in the phosphorylation of *DRP1* at SER637. SER637 as discussed above, inhibits *DRP1* activity, so a reduction in phosphorylation at SER637 is followed by an increase in mitochondrial fragmentation and a reduction in mitochondrial membrane potential (Gawlowski et al., 2012). Another more recent study on O-GlcNAcylation of *DRP1* discussed the possibility that amyloid-beta regulates mitochondrial fission in neuronal cells (Park et al., 2021). Nitric oxide is also known to be a regulator of mitochondrial fission and is an important signaling molecule which can mediate neuronal injury when in excess. It is produced as a byproduct of the amyloid-beta protein *via* S-nitrosylation of *DRP1* (SNO-*DRP1*) (Cho et al., 2009). This study revealed that SNO-*DRP1* increased in the brains of humans with Alzheimer’s disease, leading to synaptic loss, mitochondrial fission, and neuronal damage.

## Regulation of mitochondrial motility

The mechanics behind mitochondrial motility have been best studied in the neuron and the molecular mechanisms that engage mitochondria in their motility are well established. As neurons are postmitotic cells that survive as long as the organism is alive, mitochondria need to be cycled when they are subject to dysfunction or age. Conditions of stress and impaired integrity also lead to shifts in mitochondrial motility. Regulation of mitochondrial motility is therefore incredibly important to meet the necessary metabolic requirements to adapt to stressors, as well as to remove dysfunctional and aged mitochondria while replenishing the cell’s supply of healthy ones. The implications of a defect in mitochondrial transport are seen in the pathogenesis of a multitude of major neurological disorders. The coordination between these mechanisms to distribute mitochondria in neurons and in other cells, however, is not very well understood.

The bidirectional transport of mitochondria along microtubules is carried out by kinesin and dynein motors as discussed previously, and is frequently described as a “tug of war.” Our mechanistic understanding of how motor proteins, adapters, and corresponding regulatory proteins interact at the molecular level is lacking. With the amount of research that has been done, we cannot accurately predict how disturbances at a cellular level play a role in the determination of net retrograde or anterograde movement, but there are hypotheses as to how this occurs. Much of the research done regarding how kinesins and dyneins regulate movement of a cargo being transported along a microtubule is based on mechanics and the velocity or static behavior of mitochondria are dependent on the balance of forces. Retrograde and anterograde velocity is thought to be regulated by the amount of kinesin and dynein molecules acting on a mitochondria as well as the overall load force the motors need to act against, such as the dynein stall force (Barnhart, 2016; Ohashi et al., 2019). Other factors may include specific protein-protein interactions between Snph and mitochondria (Kang et al., 2008), myosin activity opposing the axonal transport of mitochondria (Pathak et al., 2010), and even viscous drag due to high microtubule density (Wortman et al., 2014). Furthermore, the post-translational modifications of microtubules may play a role in controlling motor protein velocity and processivity (Sirajuddin et al., 2014) with a study showing that there is a possible kinesin motility bias toward the axon due to increased acetylation of axonal microtubules (Hammond et al., 2010). It still remains unclear though the extent at which post-translational modifications actually has on mitochondrial motility.

**FIGURE 3:**
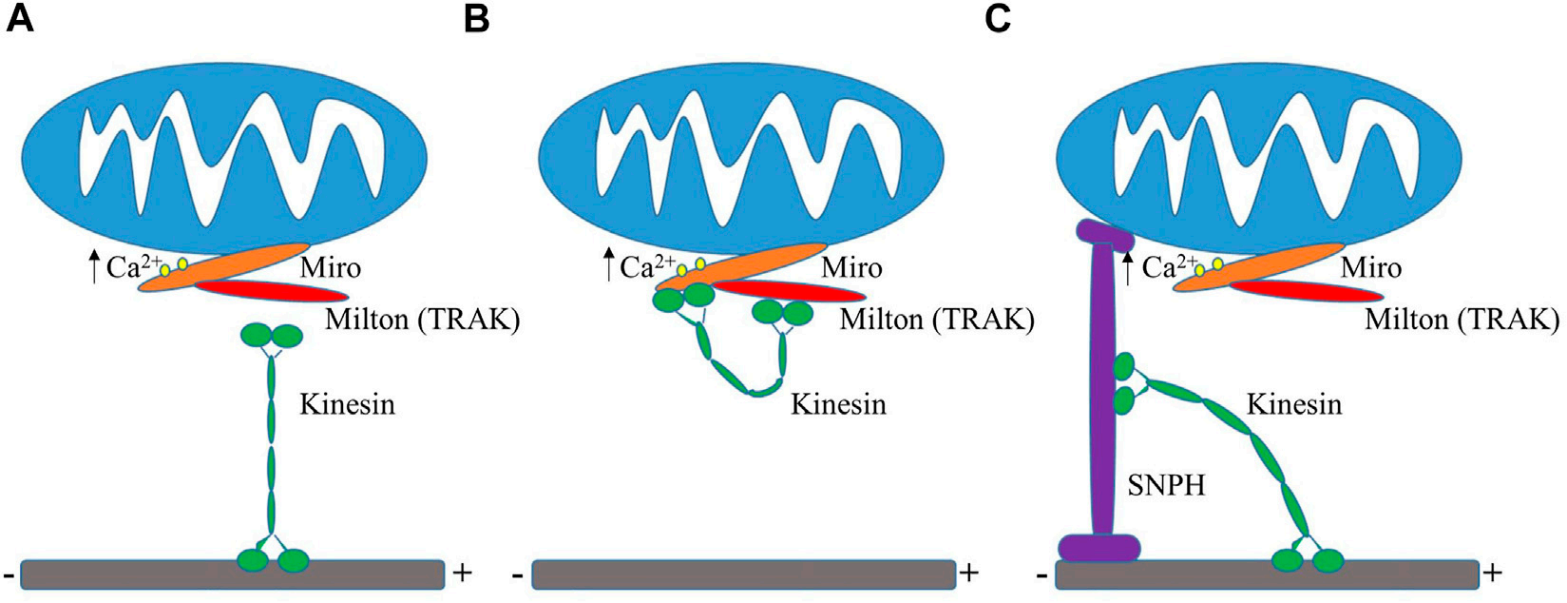
Mito-Ca2+ sensing models. **(A)** Ca^2+^ binding to Miro results in the uncoupling of mitochondria from kinesin and the possible deactivation of motors (Macaskill et al., 2009b). **(B)** High levels of Ca^2+^ uncouple kinesin machinery from the microtubule, leading to its direct binding to Miro (Wang and Schwarz, 2009). **(C)** Display of the “Engine-Switch and Brake” model in which kinesin detaches from Miro and subsequently interacts with the mitochondrial docking protein Snph (Chen and Sheng, 2013).

Mitochondrial distribution is heavily correlated with the demand for energy. Mitochondria are typically clustered at sites of high energy demand because of a higher need for ATP generation, such as near synapses or surrounding the cell nucleus (Jang et al., 2019; Green et al., 2021), which lacks its own mitochondria. Elevated intracellular Ca^2+^ levels draw mitochondria to synapses by activation of the voltage-gated calcium channels at presynaptic terminals or NMDA receptors at postsynaptic sites (Rintoul et al., 2003; Chang et al., 2006; Szabadkai et al., 2006). Studies over the past decade focused heavily on how mitochondria are recruited and arrested at synapses and new insights revealed that synaptic Ca^2+^ levels play a role in the regulation of mitochondrial trafficking and anchoring when looking into KIF5-TRAK-Miro complexes (Sheng, 2014). Miro specifically was found to serve as a regulator of mitochondrial motility by sensing Ca^2+^ levels. Miro calcium-binding *via* EF hands either inactivates or disassembles the KIF5-TRAK-Miro transport machinery arresting mitochondria at active synapses (Saotome et al., 2008; Macaskill et al., 2009b; Wang and Schwarz, 2009). These studies purport that there are two differing Mito-Ca^2+^ sensing models as of now to explain how this occurs (Figure 3). One model shows that the direct interaction between Miro and KIF5 is inhibited by the binding of calcium, which results in mitochondria uncoupling from the transport machinery and the potential halting of the molecular motors (Macaskill et al., 2009b). A second model discusses that under normal calcium levels, Miro binds to KIF5 *via* Milton while during high levels of calcium, the kinesin motor domain unbinds from the microtubule and turns upward to directly bind to Miro on the mitochondria, effectively uncoupling the mitochondria from the microtubule transport pathway (Wang and Schwarz, 2009). It has also been purported that the binding of calcium to Miro prompts a conformational change that either reduces microtubule engagement of Milton-bound kinesin or decreases the volume of kinesin molecules mitochondrially-bound (Macaskill et al., 2009b). One hypothesis is that Miro calcium sensing facilitates the localization of mitochondria to active synapses by decreasing the amount of kinesin motors actively driving motility. Miro expression elevation has also been shown to increase mitochondrial motility through the recruitment of *TRAK* and motors to mitochondria (MacAskill et al., 2009a).

The mitochondrial docking protein Snph as discussed before immobilizes axonal mitochondria. Mitochondrial movement is arrested by binding kinesin and inhibiting ATPase activity through the reduction of active kinesin motors attached to a singular mitochondrion as well as creating a greater force that any remaining motors must overcome. Snph also becomes important for calcium-induced mitochondrial arrest in axons. The mechanisms that regulate Snph-mediated mitochondrial docking are unknown for the most part but there have been some recently proposed models such as the “Engine-Switch and Brake” model where Snph serves as an engine-off switch when it senses Miro GTPase-Ca^2+^ and as a brake when it anchors mitochondria to the microtubule track (Chen and Sheng, 2013). The arrest of mitochondrial motility can also be affected by nutrient availability as seen by the increase of extracellular glucose concentrations mediated by post-translational modifications of the protein Milton by the enzyme O-GlcNAcylation transferase (Pekkurnaz et al., 2014). The GTPase Rac1 regulates VimIFs and their binding to mitochondria (Matveeva et al., 2015).

## Mitochondrial diseases and mitochondrial dynamics

As discussed earlier in this review, fission allows for the segregation of damaged mitochondria while fusion exchanges materials between damaged and functional mitochondria. The balance between fission and fusion is what allows mitochondria to remain healthy and supply enough energy to remain fully functional. Alterations to mitochondrial dynamics therefore play a critical role in the manifestation of mitochondrial disorders.

### Charcot-Marie-Tooth disease type 2A

Charcot-Marie-Tooth disease (CMT) is a rare heterogenous inherited neuropathy. Those affected experience peripheral neuropathy, hypotonia and severe progressive muscle weakness in the distal limbs resulting in gait defects. The most common type, CMT Type 2A (CMT2A), is known to be caused by defects to the inner mitochondrial membrane fusion protein, *MFN2*, and remains an incurable condition (Kijima et al., 2005; Cho et al., 2007). Current models are looking into mitochondrial transport defects as a cause for CMT2A because *MFN2* has been reported to interact with the Milton/Miro/kinesin complex in neurons (Misko et al., 2010). *MFN2* mutations associated with CMT2A have been shown to create mitochondrial clusters when overexpressed in cell cultures (Detmer and Chan, 2007). This clustering suggests that the *MFN2* mutations do indeed have an effect on mitochondrial locomotion. Transgenic mouse models overexpressing T105M or R94W mutations confirm this model as the clumping of mitochondria is associated with these phenotypes and there is a decrease in mitochondrial motility in neurons with a sparsity of action in the axons (Detmer and Chan, 2007).

The authors of one interesting study regarding the role of *MFN2* in cells concluded that the defect in locomotion was not a result of the depletion of *MFN2*, but instead due to the incorrect mitochondrial shaping. They came to this conclusion after seeing that mouse embryonic fibroblasts that were isolated from *MFN2* knockout embryos exhibited normal mitochondrial locomotion. Both short tubular and round mitochondria were present and even though the round mitochondria seemed to lose their directed movement, they retained the ability to fuse with the mitochondrial network to create mitochondria having undirected movement (Chen H. C. et al., 2003). There is a possibility that *MFN2* plays a role in quality control to ensure that only healthy mitochondria with the ability to fuse are transported (Cartoni and Martinou, 2009).

Another function of *MFN2* is its association with ER-mitochondrial tethering. Many key cellular functions occur due to this connection including lipid and calcium homeostasis and fission of mitochondria. This model is speculative as it is disputed over whether this tethering is promoted or inhibited by *MFN2* (Filadi et al., 2015; Naon et al., 2016). More studies regarding this behavior need to be conducted to establish the definitive role of *MFN2*.

### Dominant optic atrophy

DOA is considered to be the most common inherited optic neuropathy. Most cases of DOA are tied to heterozygous mutations in *OPA1*, which, as discussed previously, is necessary for IMM fusion and maintenance of OXPHOS supercomplexes and cristae membrane ultrastructure (Alexander et al., 2000; Yarosh et al., 2008). The overall lack of *OPA1* functionality leads towards increased mitochondrial fission and mitochondrial network fragmentation. This in turn increases reactive oxygen species, alters calcium homeostasis, and impairs oxidative phosphorylation. The progressive loss of retinal ganglion cells located in the inner retina and subsequent atrophy of the optic nerves cause bilateral loss of vision resulting in pallor of the optic disk (Kline and Glaser, 1979; Delettre et al., 2002).

### Mitochondrial dynamics and neurodegenerative diseases

Mitochondrial dysfunction is a prominent early marker and pathological feature for a number of neurodegenerative diseases such as Huntington’s disease (HD), Parkinson’s disease (PD), Alzheimer’s disease (AD), and amyotrophic lateral sclerosis (ALS). Abnormal mitochondrial dynamics lead to the progressive loss of structure and function of neurons within the central and peripheral nervous systems. With the discovery of fusion and fission proteins, namely *DRP1*, *OPA1*, and *MFN2*, mitochondrial dynamics regulators and their functions have been increasingly studied to try and find novel therapeutic strategies for these well-known and incurable diseases. There is a lack of research regarding mitochondrial motility and how this plays a role in the development of disorders related to mitochondrial dynamics. While difficult to observe and assess, this could be an important avenue of research that can shed more light on these diseases.

HD is an autosomal dominant neurodegenerative disease caused by the expansion of the CAG triplet nucleotide responsible for encoding a polyglutamine tract in exon 1 of the HTT (huntington) gene (Warby et al., 2009). The mutant HTT variant activates *DRP1* through the enhancement of its GTP-hydrolyzing activity (Song et al., 2011), thus causing polyglutamine accumulation and mitochondrial fragmentation. These observations are further backed up through ameliorative results in models of Huntington’s disease in which *DRP1* is inhibited or *MFN2* is augmented in a way to restore fusion preventing cell death (Wang et al., 2009). Mutant HTT has also been shown to interact with motor proteins by inactivating them or by disrupting the connection between microtubules and motor proteins resulting in mitochondrial trafficking impairment (Trushina et al., 2004).

PD has been extensively studied as it is the second most prominent neurodegenerative disease behind AD. Symptoms include bradykinesia, resting tremors, and rigidity. Mitophagy related proteins *PINK1* and *Parkin* have been linked to PD and mutations in either result in impaired mitophagy and early accumulation of *DRP1* in neurons. The excessive amount of fission leads to excessive fragmentation accompanied by oxidative stress and reduced ATP production (Dagda et al., 2009; Lutz et al., 2009). Some missense mutations in *OPA1* (G488R, A495V) have also been seen in patients with syndromic parkinsonism as well as dementia (Carelli et al., 2015).

AD experimental models display altered expressions of mitochondrial fusion and fission regulators like *DRP1*, *MFN 1* and *2*, *OPA1*, and *Fis1*; however, detailed mechanisms physically connecting mitochondrial dynamics regulators and AD have yet to be determined. There is mounting evidence regarding autophagy and its important role in the pathogenesis of AD (Zare-Shahabadi et al., 2015). This shows that abnormal mitochondrial dynamics and morphological changes may be important pathways with contributions to mitochondrial dysfunction, and by association, neuronal dysfunction in the brains of those with AD.

ALS is a rare and heritable form of disease that progressively degenerates motor neurons in the brainstem and spinal cord. ALS causes issues with muscle strength, atrophy, swallowing, difficulty in speaking, and breathing. Abnormal mitochondrial morphology as well as mitochondrial fragmentation have been documented in both ALS cell and animal models (Rodriguez et al., 2012; Peng et al., 2016b). Mutations in superoxide dismutase 1 (SOD1) have shown signs of mitochondrial fragmentation due to the changed expression of a few notable mitochondrial fission and fusion proteins such as *DRP1*, *MFN1*, *OPA1*, and *Fis1* (Vande Velde et al., 2011; Liu et al., 2013; Song et al., 2013; Vinsant et al., 2013). Miro downregulation in the spinal cords of ALS patients and mutant SOD1 and TDP-43 transgenic mice also seems to contribute to mitochondrial motility impairments prominent in ALS (Zhang et al., 2015). Despite all of this, mitochondrial fragmentation in these experimental models and patients suffering from ALS is likely to be a result, and not a cause, of the onset of the disease, given that there are no reported physical associations between ALS associated proteins and mitochondrial dynamics proteins.

### Mitochondrial dynamics and cardiovascular disease

The heart relies on mitochondria to produce enough ATP to sustain its contractile functions. Approximately 30% of the total cell volume is occupied by mitochondria alone (Hall et al., 2014). Mitochondrial dynamics are being studied more recently in association with varying fields of cardiovascular biology including cardiomyopathies (Fang et al., 2007), heart development (Martin-Fernandez and Gredilla, 2016), myocardial infarction (Davidson et al., 2014) and ischemia (Ashrafian et al., 2010).

Mitochondrial dynamic impairment represented by a decrease in mitochondrial fission as mediated by *DRP1* expression can be associated with a decrease in ATP production. In addition, a reduction in mitochondrial fission negatively impacts mitochondrial quality control, and as discussed previously, leads to an accumulation of damaged mitochondria. Quality control of mitochondria has been shown to be extremely critical when it comes to cardiovascular pathologies (Marzetti et al., 2013).

## Mitochondrial therapeutics

An emerging field of therapeutics is developing new treatment strategies to remedy mutations, dysfunctions, and diseases affecting mitochondrial biogenesis, dynamics, and healthy aging. Therapeutics targeted at mitochondrial dysfunction have the potential to make a very broad clinical impact. In particular, treatments that focus on the amelioration of derangements in mitochondrial dynamics observed in a multitude of mitochondrial diseases, have the potential to significantly impact cellular function and survival, thus markedly improving patient outcomes. As has been previously discussed, maintenance of balanced mitochondrial fusion and fission events and normal mitochondrial motility are essential to enjoying good health.

Dysfunctional mitochondria can be distinguished based on their morphology and can be degraded through selective autophagy (Dufour et al., 2011). Pro-autophagic agents have been used as a therapy in recent years. Rapamycin, an inhibitor of the mammalian target of rapamycin (mTOR) pathway, and its derivatives are among the most studied therapeutic agents for the treatment of neurodegenerative diseases, including PD and HD (Sarkar et al., 2008; Johnson et al., 2013; Villa-Cuesta et al., 2014). Some recent studies showcase positive results of rapamycin intervention in LS mouse and *Drosophila* models (Li Q. et al., 2014; Wang et al., 2016). The pharmacological inhibition of TOR rescues the shortened lifespan, neurodegeneration, and neurological symptoms in the mouse models of LS. Using the *Drosophila* model, investigators found that the increased lifespan occurred in an autophagy-independent manner. The authors of these papers concluded that the improvement could potentially have been the result of an immunosuppressive effect of the rapamycin as opposed to a direct effect on mitochondrial function. More research is needed to discern any direct effects that rapamycin and related autophagic agents have on mitochondrial dysfunction.

Another popular strategy for mitochondrial disease therapy is the use of pharmacological agents to inhibit mitochondrial fission. The inhibition of mitochondrial fission proteins *DRP1*/*Fis1* has been shown to be reduce excessive mitochondrial fission and heart damage in cultured murine cardiac myocytes from whole rat heart models of ischemia and reperfusion (IR) injury (Sharp et al., 2014). Use of fission inhibitors Mdivi-1, calcineurin inhibitor, DRP1 siRNA, and therapeutic hypothermia were shown to reverse mitochondrial swelling and fragmentation in these IR models. The application of these small molecules that inhibit mitochondrial fragmentation are important therapeutic factors to be further studied because uncontrolled mitochondrial fusion is a significant contributor to a number of neurodegenerative diseases (Chen and Chan, 2009). Mdivi-1, a *DRP1* inhibitor, and the selective peptide inhibitor P110 have also been reported to rescue mitochondrial morphological and functional defects induced by mutations in *PINK1* (Qi et al., 2013). The continual study of small molecule mitochondrial fission inhibitors may be useful in the treatment and prevention of mitochondrial disorders.

Some lifestyle interventions that are recognized to have promising effects are regular exercise and caloric restriction. Many studies have shown that exercise has an effect on mitochondrial fission and fusion as well as mitochondrial biogenesis. Mitochondrial biogenesis and turnover rates decrease as organisms age causing a myriad of health problems. Exercise has become recognized as an intervention to increase mitochondrial biogenesis as well as carbohydrate levels and fatty acid oxidation in skeletal muscle (Holloszy and Booth, 1976). Exercise leads to an increase in stress signals which appear to be responsible for activation of mitochondrial biogenesis post-exercise. These signals include increased levels of cytosolic Ca^2+^ (Baar et al., 2003), AMP/AMPK (McConell et al., 2010), and ROS (Irrcher et al., 2009). Exercise also stimulates PGC-1a, a transcription coactivator. Deficiency shows blunted expression of genes for oxidative phosphorylation and decreased mitochondrial function (Zechner et al., 2010).

Caloric restriction (CR) is an effective nutritional intervention that helps in the prevention of age-related metabolic disorders (Omodei and Fontana, 2011). The molecular mechanisms of CR-induced benefits are still under further investigation. Many studies have demonstrated that CR reduces the overproduction of ROS and oxidative damage (Barja and Herrero, 2000; Barja, 2002) which leads to the enhancement of mitochondrial function in humans. CR also increases mitochondrial biogenesis through the activation of PGC-1a, a key regulator of energy metabolism (Lopez-Lluch et al., 2008).

Other pharmacological interventions for mitochondrial disorders include mitochondria-targeted antioxidants and a CR mimetic, resveratrol, which has been demonstrated to affect mitochondrial biogenesis in liver, muscle, and brain. Resveratrol has also been shown to promote the expression of proteins involved in mitochondrial fission and fusion resulting in a protective effect in mitochondria (Peng et al., 2016b). Oxidative damage from mitochondria is associated with numerous metabolic disorders and antioxidant therapies pose a potential route for treatment. In the past few years, antioxidant compounds containing ubiquinone (MitoQ) or vitamin E specifically targeted to mitochondria have been used in the treatment of mitochondrial dysfunctions (Kelso et al., 2001). Some recent studies have shown it to be effective by inhibiting oxidative stress and apoptosis (Chen et al., 2020) and the restoration of mitochondrial respiration in heart failure induced by pressure overload (Ribeiro Junior et al., 2018). These pharmacological interventions are not directly tied to mitochondrial dynamics and are more so related to mitochondrial membrane potential and relieving oxidative stress making them important for mitochondrial health.

## Discussion

Mitochondria are complex and dynamic intracellular organelles that undergo highly regulated motile interactions involving numerous complexes (e.g., kinesins, cytoskeletal elements). They are also subject to fusion and fission events under the control of regulating proteins. Mitochondrial fission and fusion maintain the functionality of mitochondria and plays an incredibly important role in cell fate. Fission is responsible for proper distribution and number of mitochondria while fusion ensures the exchange of content for optimal mitochondrial activity. Disruption of mitochondrial motility or an imbalance in mitochondrial fusion/fission is associated with organ dysfunction and diseases known to involve the neurological, vascular, and skeletal systems. With an evolving understanding of mitochondrial dynamics, some therapeutic avenues are being researched to combat dysfunctional dynamics with many trying to reverse excess fission (Mdivi-1, calcineurin inhibitor, etc.). There is a lack in knowledge of the molecular events involved in mitochondrial fusion and fission, requiring more mechanistic understanding. Further studies regarding how fusion and fission is related to mitophagy is promising as it relates to mitochondrial disorders and cancer. The field of mitochondrial dynamics is expanding and more research regarding mitochondrial motility and its role in the pathogenesis of disease states and dysfunction is necessary. An expanding pool of experimental techniques and imaging tools offer potential for novel discoveries in mitochondrial dynamics understanding as well as novel treatment approaches for mitochondrial diseases involving errors of dynamic functions.
